# High production of ectoine from methane in genetically engineered *Methylomicrobium alcaliphilum* 20Z by preventing ectoine degradation

**DOI:** 10.1186/s12934-024-02404-2

**Published:** 2024-05-02

**Authors:** Sang Eun Lim, Sukhyeong Cho, Yejin Choi, Jeong-Geol Na, Jinwon Lee

**Affiliations:** 1 Department of Chemical and Biomolecular Engineering, Sogang University, Seoul, Republic of Korea; 2 C1 Gas Refinery R&D Center, Sogang University, Seoul, Republic of Korea

**Keywords:** Methane, Methanotroph, Ectoine, *Methylomicrobium alcaliphilum* 20Z, Nitrogen source

## Abstract

**Background:**

Methane is a greenhouse gas with a significant potential to contribute to global warming. The biological conversion of methane to ectoine using methanotrophs represents an environmentally and economically beneficial technology, combining the reduction of methane that would otherwise be combusted and released into the atmosphere with the production of value-added products.

**Results:**

In this study, high ectoine production was achieved using genetically engineered *Methylomicrobium alcaliphilum* 20Z, a methanotrophic ectoine-producing bacterium, by knocking out *doeA*, which encodes a putative ectoine hydrolase, resulting in complete inhibition of ectoine degradation. Ectoine was confirmed to be degraded by *doeA* to N-α-acetyl-L-2,4-diaminobutyrate under nitrogen depletion conditions. Optimal copper and nitrogen concentrations enhanced biomass and ectoine production, respectively. Under optimal fed-batch fermentation conditions, ectoine production proportionate with biomass production was achieved, resulting in 1.0 g/L of ectoine with 16 g/L of biomass. Upon applying a hyperosmotic shock after high–cell–density culture, 1.5 g/L of ectoine was obtained without further cell growth from methane.

**Conclusions:**

This study suggests the optimization of a method for the high production of ectoine from methane by preventing ectoine degradation. To our knowledge, the final titer of ectoine obtained by *M. alcaliphilum* 20ZDP3 was the highest in the ectoine production from methane to date. This is the first study to propose ectoine production from methane applying high cell density culture by preventing ectoine degradation.

**Supplementary Information:**

The online version contains supplementary material available at 10.1186/s12934-024-02404-2.

## Background

Methane is a major greenhouse gas with a global warming potential at least 20 times higher than that of carbon dioxide [1]. Atmospheric methane concentrations have been steadily increasing and exceeded 1,900 parts per billion in 2022 [2]. Recently, as the industry’s interest in methane emissions has grown, the “Global Methane Pledge” was declared, marking the beginning of heightened attention towards methane reduction. Although technologies exist for the chemical conversion of methane to liquid fuel, they are inefficient owing to the cost and complexity of the process and the extreme reaction conditions such as high temperature and high pressure [3, 4]. Thus, the biological conversion of methane, which is relatively simple and operates at an ambient temperature and pressure, has been proposed as an alternative [5]. Methane is also highly valued as a next-generation carbon source because it is relatively cheaper than sugar or its derivatives, and does not compete with food resources [6].

Methanotrophs are promising biocatalysts for the production of chemicals such as polyhydroxyalkanoates, single-cell proteins, and methanol from methane [7–9]. However, the production of these methane-derived bulk chemicals often cannot compete with petrochemical products in terms of price, due to the low gas-to-liquid mass transfer, low productivity of processes, leading to high investment and operational costs [10–12]. In this context, the production of ectoine, an osmotic protector with a retail market value around 1,000 $ kg^− 1^ and an annual demand of 20,000 tons, has recently attracted increasing attention of industries producing fine chemicals from methane [13]. Ectoine, which protects cells, enzymes, proteins, and other biomolecules, is primarily used in cosmetics and biomedical applications [14, 15]. Recent studies have reported that high profitability of the process from methane to ectoine comparing the production costs of ectoine and the current market price of this chemical [12, 16].

**Fig. 1:**
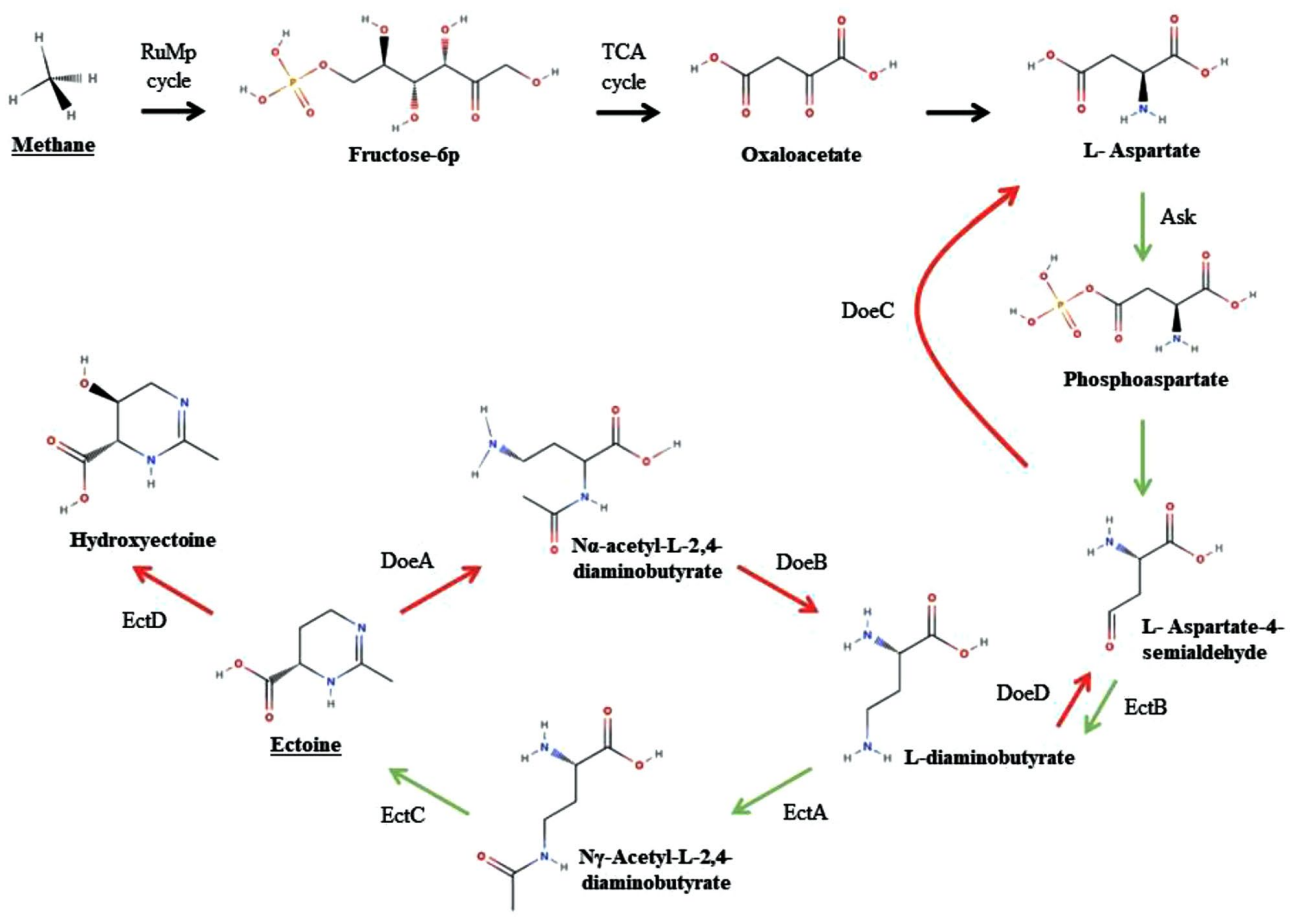
Metabolic pathway for ectoine bio-synthesis and degradation in *M. alcaliphilum* 20Z. Ask: aspartate kinase; AsdB: aspartate-semialdehyde dehydrogenase; EctB: L-2,4-diaminobutyrate transaminase; EctA: L-2,4-diaminobutyric acid acetyltransferase; EctC: L-ectoine synthase; EctD: ectoine hydroxylase; DoeA: ectoine hydrolase; DoeB: N2-acetyl-L-2,4-diaminobutanoate deacetylase; DoeC: aspartate-semialdehyde dehydrogenase; DoeD: L-2,4-diaminobutyrate transaminase

*Methylomicrobium alcaliphilum* 20Z is a methanotrophic ectoine-producing bacterium. Its full genome sequence has been identified and relevant genetic tools have been established [17]. *M. alcaliphilum* 20Z has a pathway for ectoine synthesis by the sequential action of Ask, EctB, EctA, and EctC as described in Fig. 1 [18]. Ectoine is converted into hydroxyectoine by EctD in *M. alcaliphilum* 20Z. But, also ectoine degradation pathway exists in *M. alcaliphilum* 20Z mediated by DoeA, DoeB [19].

The main process used for ectoine production is sugar fermentation by *Halomonas* species [20, 21]. However, this process is costly because it requires high-quality carbon sources, such as glucose, sodium glutamate, and yeast extract [15]. Although the reported production of ectoine using methanotrophs is much lower than that achieved with traditional sugar-fermenting microbes, the combined process of ectoine production and the treatment of atmospheric methane can reduce the costs associated with ectoine production while also promoting climate change mitigation through active methane reduction. Thus, methanotrophs provide an excellent environmental benefit of producing ectoine from dilute methane.

This study suggests the optimization of a method for enhanced production of ectoine from methane by knocking out *doeA*, a putative ectoine-degrading hydrolase gene, from *M. alcaliphilum* 20ZDP2, which was constructed by disrupting *ectD* and *ectR* genes. Notably, it was confirmed that the nitrogen source is crucial for ectoine synthesis, with production ceasing once the nitrogen source in the medium was depleted. This emphasizes the need for continuous replenishment of nitrogen source in the medium to maintain ectoine production concurrent with cell growth. To the best of our knowledge, the final titer of ectoine obtained from *M. alcaliphilum* 20ZDP3 via this process is the highest reported ectoine production from methane to date. This is the first study to propose ectoine production from methane applying high–cell–density culture by preventing ectoine degradation.

## Materials and methods

### Bacterial strains and culture media

Halophilic, methanotrophic *Methylomicrobium alcaliphilum* 20Z was used for ectoine production. Specifically, the *ectD* and *ectR* gene–deficient 20ZDP2 strain was constructed in our previous study [22] and 20ZDP3, 20ZDP4, and 20ZDP5 defective in *doeA, doeD, ectB* genes, respectively, from 20ZDP2 were constructed in this study (Table 1). The bacteria were cultured in a modified Methylomicrobium medium described in the previous study comprising 30% (v/v) methane or 9.93 g/L methanol [22]. The strains were cryopreserved with 10% (v/v) dimethylsulfoxide added in the early exponential phase.

**Table 1:** Strains and vectors used in this study

	Characteristics	References or source
Strains		
*Escherichia coli* DH10B	F- *mcrA* ∆(*mrr-hsdRMS-mcrBC*) ϕ80*lac*Z∆M15 ∆*lac*X74 *rec*A1 *end*A1 *ara* ∆139 ∆(*ara, leu*)7697 *gal*U *gal*K λ- *rps*L (StrR) *nup*G	RBC Bioscience
*Escherichia coli* S17-1 λ*pir*	Donor strain	
*Methylomicrobium alcaliphilum* 20Z	host strain	DSMZ
*M. alcaliphilum* 20ZDP2	*M. alcaliphilum* 20ZDP Δ*ectD*Δ*ectR*	[22]
*M. alcaliphilum* 20ZDP3	*M. alcaliphilum* 20ZDP Δ*ectD*Δ*ectR*Δ*doeA*	This study
*M. alcaliphilum* 20ZDP4	*M. alcaliphilum* 20ZDP Δ*ectD*Δ*ectR*Δ*doeD*	This study
*M. alcaliphilum* 20ZDP5	*M. alcaliphilum* 20ZDP Δ*ectD*Δ*ectR*Δ*ectB*	This study
**Vectors**		
pK19mobsacB	suicide vector for gene deletion	
pK19mobsacBΔ*doeA*	pK19mobsacB containing flank regions of *doeA*	This study
pK19mobsacBΔ*doeD*	pK19mobsacB containing flank regions of *doeD*	This study
pK19mobsacBΔ*ectB*	pK19mobsacB containing flank regions of *ectB*	This study

### Deletion of ectoine-degradation genes from *M. Alcaliphilum* 20ZDP2

Deletion of *doeA, doeD*, or *ectB* from *M. alcaliphilum* 20ZDP2 was performed with suicide vectors using the conjugation method described previously [22, 23]. The flanking regions of the target gene were cloned into a suicide vector, pK19mobsacB, after restriction digestion using *Hin*d III and *Eco*RI using an infusion system (In-Fusion™ HD Cloning Kit, Takara Bio Inc., Japan). All subsequent procedures, such as the conjugation of vectors to *M. alcaliphilum* 20ZDP2 and sucrose counterselection for *doeA* or *ectB* deletion, were carried out as previously described [22]. The knock–out of target genes was confirmed by PCR. All the oligonucleotides used in this study are listed in Table 2.

**Table 2:** Oligonucleotides used in this study

Oligonucleotides	Sequence
Upstream of *doeA* F/R	tgacatgattacgccaagcttatgaaagtctttgaacaatgggaa/ccctccttgcaaggcctccaaagcatccg
Downstream of *doeA* F/R	Tggaggccttgcaaggagggttactcaatggc/aaaacgacggccagtgaattcttaacccagccaaacctgctgc
Integration to upstream of *doeA* F/R	atgcttccggctcgtatgtt / tccggatcgctgattacgac
Integration to downstream of *doeA* F/R	gcgtcgaaggcgataaaacc / taagcccactgcaagctacc
Confirmation of *doeA* deletion F/R	gcttcgaacgcggactacta / cgctgttgaggccgtaagtt
Upstream of *doeD* F/R	tgacatgattacgccaagcttatggccatacagtgggatcagc/ gttcaatcatgaatactctccttacggttgacagg
Downstream of *doeD* F/R	gagagtattcatgattgaacgcgacgacatg/ aaaacgacggccagtgaattcctaagccccgtattcgggt
Integration to upstream of *doeD* F/R	cagtgagcgcaacgcaatta / cgcctgcgaagaagtcgata
Integration to downstream of *doeD* F/R	gccttggcgaaaaacatcgt / ggacaggtcggtcaatcgtt
Confirmation of *doeD* deletion F/R	cgattgagagatgtcggggg / cggtatcgggataaggtcgc
Upstream of *ectB* F/R	tgacatgattacgccaagctttcaggacgcgaggcatattgc/ gagaattagaagcccgctgagccaaccag
Downstream of *ectB* F/R	tcagcgggcttctaattctctcctgagcaagatgg/aaaacgacggccagtgaattcttaaaccggaaaatcaaacgc
Integration to upstream of *ectB* F/R	ttgccgtcaggtgaaacgat / catgggctctgtttgagggg
Integration to downstream of *ectB* F/R	ccgggtgattgtgaccgtaa / taagcccactgcaagctacc
Confirmation of *ectB* deletion F/R	atgcatgcgaaaatcggcac / ggcagtgctgtgatgtttga
RT-PCR for 16s rRNA gene F/R	tcccgggccttgtacacacc / gtggtaagcgccctcccgaa
RT-PCR for *doeA* gene F/R	ggcgtcgaaggcgataaaac/ cccgtattcgggtttcacca
RT-PCR for *doeB* gene F/R RT-PCR for *doeD* gene F/R	ctcacgaattcacacgcgac / tgcatcaatgtccggcgata gccttggcgaaaaacatcgt / caaagcatccgctaagacgc

### Batch culture in a flask

For flask fermentation, seed culture was carried out for 2 days in Methylomicrobium medium containing 3% (w/v) NaCl and 9.93 g/L methanol with the cryo-preserved cell stock at 30 °C and 230 rpm. For the main culture, culture broth was inoculated at an initial OD_600 nm_ of 0.2 to 50 mL medium containing 6% (w/v) NaCl and 0.05 µM tungsten in a 250-mL baffled flask. The headspace of the flask was filled with 30% (v/v) methane gas mixed with air and refreshed every 12 h using a mass flow controller (GMC 1200-MMOO-O-1, ATOVAC, Korea). Flask culture was conducted at 30 °C and 230 rpm and samples taken every 24 h to analyze cell growth and ectoine production. All batch fermentations were performed in triplicate.

### Fed-batch culture in a 5-L bioreactor

The pre-culture process was performed in the same manner as that for flask culture. All trials for ectoine production with continuous methane supply and pH control were performed in a 5-L stirred bioreactor (Bio Control & System, Korea) with a working volume of 3 L. All cultivations were conducted at 30 °C, and the pH was maintained at 8.9 to 9.1 using 2.5 M H_2_SO_4_ and 5 M NaOH. Air with 30% methane was supplied at 0.1–0.52 vvm with an agitation speed of 300–650 rpm for controlling the dissolved oxygen (DO) levels. To increase biomass, the DO level was maintained at 20% by controlling the agitation speed and flow rate of the gas. The initial OD_600 nm_ value was 0.2; further, 1X trace elements and 20 mM KNO_3_ were added when the dry cell weight (DCW) increased by 2 g/L.

### Accumulation of ectoine after high–cell–density culture

To accumulate ectoine by a hyperosmotic shock, 30 g/L of NaCl was added to the medium (the final salinity reached was 6% (w/v)) when the OD_600 nm_ value reached 110 (22 g/L of DCW) in a bioreactor. Further, 5 g/L of KNO_3_ was supplied and the agitation speed and gas flow rate were decreased to 300 rpm and 0.1 vvm, respectively, to minimize the shear stress affecting cells.

### qRT- PCR

*M. alcaliphilum* 20ZDP2 was cultured for 72 h in a medium containing 1 g/L or 5 g/L KNO_3_ with methane. Total RNA was prepared using the TRIzol™ Plus RNA Purification Kit (Thermo Fisher Scientific, United States). Real-time PCR was performed after cDNA synthesis using the Thermal Cycler (Takara Bio Inc., Japan) using the TB Green^R^ Premix Ex Taq™ II kit (Takara Bio Inc., Japan). The transcriptional level of ectoine-degrading genes (*doeA, doeB, doeD*) was normalized using 16 S rRNA as a housekeeping gene. Data were analyzed using the 2^−ΔΔCT^ method with the *doeA* (1 g/L KNO_3_) sample as the standard.

### Analytical procedure

Cell growth was estimated by optical density at 600 nm using a UV-VIS spectrophotometer (Biochrom WPA Lightwave II, Biochrom Ltd., UK). The DCW (g/L) was calculated as illustrated in a previous study [24].

For the quantitative analysis of intracellular ectoine, 2 mL of culture broth was harvested over time, and the cells were freeze-dried for 2 days using a freeze dryer (TFD8503, iLShinBioBase, Korea). Intracellular ectoine was then extracted from freeze-dried cells as previously reported [25] and measured using HPLC (RID-20 A, SHIMADZU, Japan) with a UV detector using the ZORBAX-NH2 column (NH2 Analytical HPLC Column 4.6 × 250, Agilent Technologies, United States) under following conditions: mobile phase, 70% (v/v) acetonitrile; temperature of column oven, 35 °C; flow rate, 0.8 mL/min.

For quantitative and qualitative analysis of the produced ectoine in fed-batch fermentation, LC-MS analysis was performed using LC (UltiMate 3000, Thermo Fisher Scientific, United states)-MS(EVOQ QUBE, Bruker, United States) with an MS/MS detector using C18 column (C18 50 × 2.1 mm, 1.9 μm, ACME) under the following conditions: mobile phase, 80% (v/v) methanol containing 0.1% (v/v) formic acid ; temperature of column oven, 30 °C; flow rate, 0.2 mL/min. This analysis was performed at The Core Facility Center for Chronic and Metabolic Diseases at Sookmyung Women’s University.

For analyzing anionic and cationic ions in the medium at various time intervals, the culture broth was centrifuged (Smart R17 Plus, HANIL SCIENCE CO., LTD, Korea) and the supernatant was analyzed using Ion Chromatography (Dionex™ Aquion™ IC System, Thermo Fisher Scientific, United States) [26].

## Results and discussion

### Development of *M. Alcaliphilum* 20Z mutants defective in ectoine degradation

In a previous study, intracellular ectoine was confirmed to be increased until the mid-exponential phase but rapidly decreased in *M. alcaliphilum* 20Z despite cell growth [18, 22, 27]. To prevent ectoine degradation after the mid-exponential phase, the genes encoding ectoine hydrolase (*doeA*) were selected for deletion. Thus, we knocked out the *doeA* gene from *M. alcaliphilum* 20ZDP2 (Δ*ectD*Δ*ectR*), which was constructed in our previous study [22]. Deletion of the *doeA* gene in *M. alcaliphilum* 20ZDP2 was confirmed via PCR (Fig S1a), and the resulting strain was named *M. alcaliphilum* 20ZDP3 (Δ*ectD*Δ*ectR*Δ*doeA*).

**Fig. 2:**
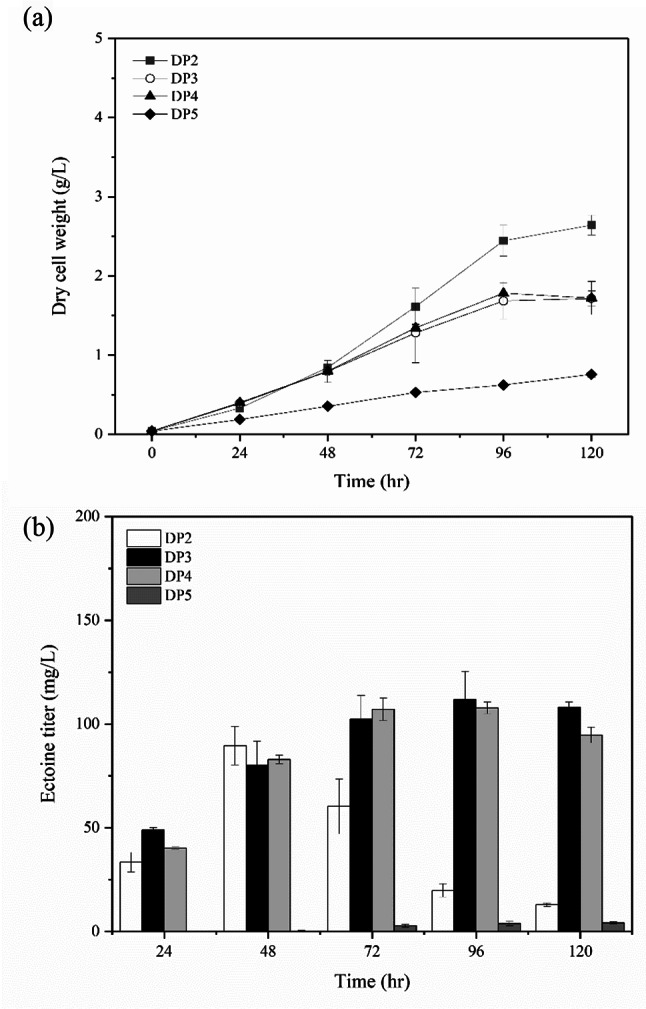
Flask culture of the mutant defective in *doeA* (*M. alcaliphilum* 20ZDP3), defective in *doeD* (*M. alcaliphilum* 20ZDP4), defective in *ectB* (*M. alcaliphilum* 20ZDP5), and *M. alcaliphilum* 20ZP2 as a control. The biomass (**a**) and ectoine (**b**) production

To investigate the effect of DoeA, flask culture was carried out with *M. alcaliphilum* 20ZDP2 and the newly constructed *M. alcaliphilum* 20ZDP3. Intracellular and extracellular ectoine levels were measured; however, extracellular ectoine was barely detectable under these conditions. The time-course cultivation profile of each strain is shown in Fig. 2. Until 48 h, cell growth and ectoine production was similar in DP2 and DP3; however, after 72 h, their production trends were very different. The biomass (DCW) in DP3 was 1.7-fold lower than that in DP2 at 72 h. In contrast, DP3 produced ectoine up to 102 mg/L for 72 h and maintained it without degradation, whereas DP2 produced ectoine up to 90 mg/L for 48 h, which then rapidly decreased. These results demonstrate that the ectoine can be degraded by DoeA and re-used for synthesizing cell constituents. Therefore, not only was the maximum ectoine production improved by up to 1.2-fold, but ectoine degradation that occurred after the mid-exponential phase was also completely prevented by disrupting the *doeA* gene.

In *M. alcaliphilum* 20Z, ectoine is synthesized by the sequential action of EctB, EctA, and EctC (Fig. 1) [17, 18]. These three genes are organized on the *ectABC*-ask operon, and the transcriptional regulation of these ectoine synthesis genes by EctR has been identified and described [28]. Notably, two genes encoding L-2,4-diaminobutyrate transaminase are present on the chromosome of *M. alcaliphilum* 20Z, as indicated in Fig. 1. One is located within the *ectABC* operon, and the other is located upstream of the *doeA* gene. Reshetnikov et al. reported that *M. alcaliphilum* 20Z possesses the putative *doeA, doeB, doeC* and *doeD* genes, which code for the enzymes involved in ectoine degradation, and these genes are aligned in the same direction, forming the *doeBDAC* cluster [19]. And it was demonstrated that DoeA and DoeB are directly involved in the hydrolysis of ectoine in *M. alcaliphilum* 20Z through the deletion of each gene. As there have been no studies on the effect of *ectB* or *doeD* deletion on ectoine synthesis and degradation, *M. alcaliphilum* 20ZDP4 (DP2Δ*doeD*) and *M. alcaliphilum* 20ZDP5 (DP2Δ*ectB)* were constructed (Table 1).

Successful deletion of *doeD* and *ectB* was confirmed by PCR for 20ZDP4 and 20ZDP5 strains, respectively (Fig S1b, Fig S1c). Flask fermentation was carried out using these newly constructed strains. As shown in Fig. 2b, the ectoine titer reached 108 mg/L at 96 h and was maintained in the DP4 strain. Interestingly, the trends in ectoine production and cell growth in DP4 were very similar to that in DP3. The observation of similar effects following the removal of either *doeA* or *doeD* may be attributed to these enzymes sequentially catalyzing the conversion of ectoine to aspartate in the metabolic pathway. In contrast, both ectoine production and cell growth were severely inhibited by deletion of the *ectB* gene from *M. alcaliphilum* 20DP2, suggesting that inhibition of ectoine production suppressed cell growth under hyperosmotic conditions. These results clearly indicated that *doeD* exhibits high activity opposite to that of *ectB*, and might be involved in ectoine degradation. Consequently, *ectB* is essential for ectoine production, whereas *doeD* contributes to ectoine degradation. More importantly, disruption of *doeA* or *doeD* blocked ectoine degradation, consequently increasing the ectoine titer. Although the ectoine production and cell growth trends were similar between DP3 and DP4, slight degradation of ectoine at 120 h was found in DP4 (Fig. 2b). Therefore, *M. alcaliphilum* DP3, which completely prevented ectoine degradation, was selected as the final ectoine producer in this study.

### Effect of copper on cell growth in batch culture

**Fig. 3:**
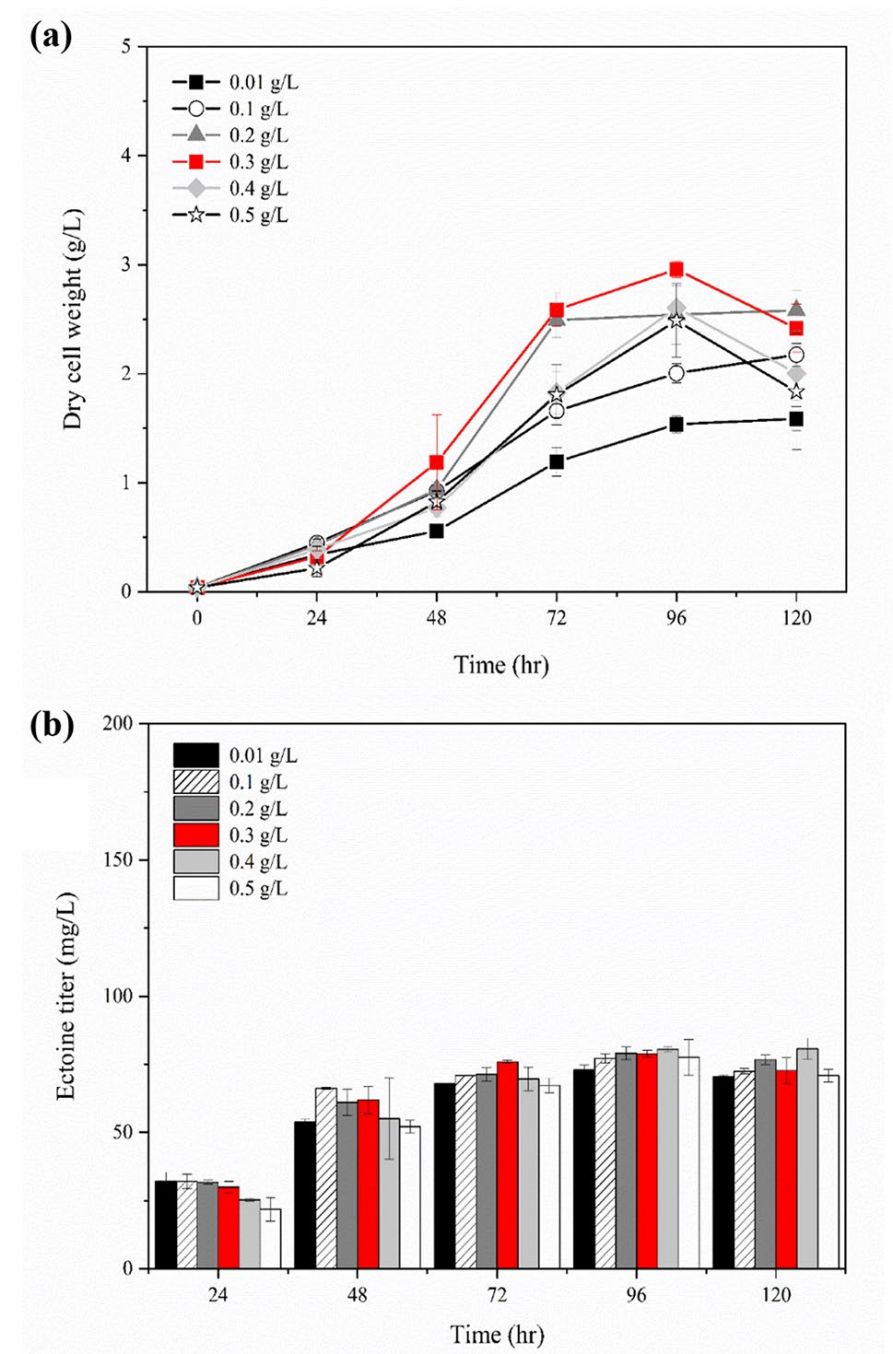
Effect of copper (Cu) addition on cell growth and ectoine production in *M. alcaliphilum* 20ZDP3. Time course of the biomass (g/L) (**a**) and ectoine (mg/L) (**b**) production from methane by *M. alcaliphilum* 20ZDP3 cultured in Methylomicrobium medium containing 6% (w/v) NaCl with 0.01, 0.1, 0.2, 0.3, 0.4, and 0.5 g/L of CuCl_2_, respectively

During batch culture of the newly constructed strain DP3, the ectoine titer was maintained with no degradation, regardless of cell growth, unlike in the DP2 strain, which degraded ectoine after 48 h of culture when approximately 1 g/L of biomass (DCW) was produced. However, after 72 h of culture, the growth rate of the DP3 strain started to slow down compared to that of DP2, resulting in a 1.7-fold lower biomass production. To improve ectoine production by stimulating DP3 cell growth, the optimal concentration of copper, a micromineral that affects cell growth, was determined. Type I methanotrophs, including *M. alcaliphilum* 20Z, use copper as a cofactor of particulate methane monooxygenase (pMMO), which mediates the first step of methane oxidation and converts methane to methanol; many studies have investigated the effects of copper addition on cell growth [29, 30]. Hence, the optimal concentration of copper (0.01–0.5 g/L) was investigated to increase cell growth. Maximum cell growth was achieved with the addition of 0.2, and 0.3 g/L CuCl_2_; however, the growth rate and biomass production were inhibited when more than 0.4 g/L of CuCl_2_ was added (Fig. 3a). In contrast, ectoine production was almost identical in all experimental groups regardless of copper concentration and gradually increased as the cells grew up to 48 h, and maintained the ectoine level without degradation (Fig. 3b). These results indicate that addition of copper to the medium might benefit cell growth by promoting methane oxidation but does not affect ectoine production.

### Effect of nitrogen source on biomass and ectoine synthesis in a batch culture

Considering that ectoine was no longer produced by DP3 strain after 72 h (Figs. 2 and 3), we presumed that the specific nutrients necessary for ectoine biosynthesis were depleted at that point. To investigate the nutrients required for increase of ectoine production in the DP3 culture, flask culture was performed with *M. alcaliphilum* 20ZDP3 in an optimized medium with methane for 120 h. The main components of the medium, such as K^+^, Mg^2+^, NO_3_^−^, and PO_4_^3−^, were analyzed using ion chromatography (Fig S1). The concentrations of K^+^, Mg^2+^, and PO_4_^3−^ were almost constant during culture; however, NO_3_^−^, the only nitrogen source, was completely depleted when the biomass (DCW) reached about 1 g/L (Fig. 2 and Fig S1). Given that the point of complete NO_3_^−^ depletion coincides with the point at which ectoine is no longer produced in the DP3 strain, a sufficient supply of nitrogen sources may be necessary for increased ectoine biosynthesis. In addition, the lower cell growth rate in the DP3 strain compared to that in DP2 at the time of complete depletion of KNO_3_ after 48 h might be caused by the failure of DP3 in degrading ectoine for reuse as a nitrogen source (Fig. 2 and Fig S1). Nevertheless, as DP3 could grow under nitrogen-starvation conditions, this strain was considered capable of fixing nitrogen in air and supplying it for cell growth, but not at sufficient levels for ectoine synthesis. *Methylomicrobium* is reported to demonstrate the potential to utilize atmospheric nitrogen gas based on identification of the *nif* gene cluster and MoFe-containing nitrogenase activity [31–35].

**Fig. 4:**
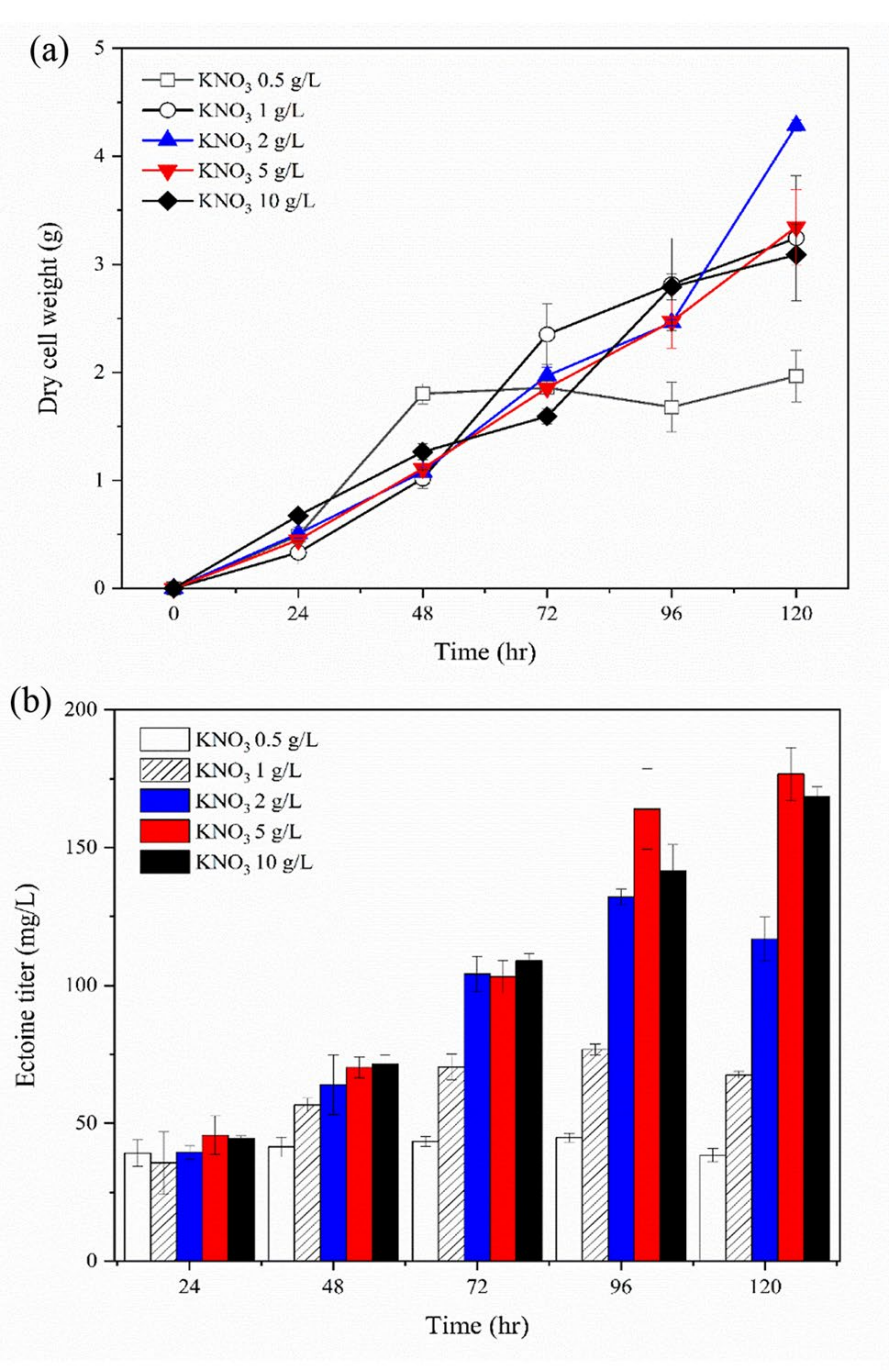
Comparison of biomass and ectoine production according to the addition of KNO_3_ in the medium. Biomass (**a**) and ectoine (**b**) production by *M. alcaliphilum* 20ZDP3 cultivated in a medium with 0.5, 1, 2, 5, and 10 g/L of KNO_3_, respectively

To evaluate the effect of nitrogen sources on the ectoine and biomass production by *M. alcaliphilum* 20ZDP3, flask culture was conducted using an optimized medium containing various concentrations of KNO_3_ (0.5, 1, 2, 5, and 10 g/L). As shown in Fig. 4, the final ectoine titer increased as the amount of KNO_3_ increased, reaching 176.7 mg/L when 5 g/L KNO_3_ was added, which was 2.3-fold higher than that obtained with 1 g/L of KNO_3_. This indicates that KNO_3_ plays a key role in ectoine biosynthesis. Meanwhile, the biomass production in all experimental groups, including that with 5 g/L KNO_3,_ was similar except for that with 0.5 g/L KNO_3_. Thus, the biomass did not increase with the concentration of KNO_3_ in the medium, and the cells could grow through nitrogen fixation from air, even if the nitrogen source was exhausted. When 0.5, 1, and 2 g/L of KNO_3_ was supplied, the ectoine titer did not show any increase although biomass was produced up to 0.5, 1, and 2 g/L, respectively. Thus, the point at which the nitrogen source was exhausted and the point at which ectoine was no longer increased coincided exactly (Fig. 4), consistent with the calculation of the amount of KNO_3_ required to support cell growth and ectoine production [36]. In other words, 1 g/L of KNO_3_ per 1 g of dry cells was required for ectoine production proportional to cell growth, maintaining the yield (mg/g DCW) of ectoine as the cells grow. When the nitrogen source was abundant, biomass and ectoine were synthesized together; however, when the nitrogen source was depleted, biomass was produced by the fixation of atmospheric nitrogen gas; however, ectoine, a nitrogen sink, was no longer produced. Further, various nitrogen sources, such as NO_2_^−^, NH_4_^+^, and urea, have been used to cultivate cells for identifying a nitrogen source useful for cell growth and ectoine production. However, *M. alcaliphilum* 20ZDP3 could only use nitrate (NO_3_^−^) as a nitrogen source (data not shown).

We hypothesized that the expression level of ectoine-degrading proteins in *M. alcaliphilum* 20ZDP2 might be regulated by depletion of the KNO_3_. To confirm that the increase in ectoine was caused by low expression levels of ectoine-degrading proteins, such as *doeA, doeB*, and *doeD* according to the supply of nitrogen source, the transcriptional level of ectoine-degrading genes was estimated by qRT-PCR in the *M. alcaliphilum* DP2 strain cultivated in medium containing 1–5 g/L of KNO_3_. To determine the effect of nitrogen starvation on the transcriptional level of related genes, the cells were harvested at 72 h when ectoine would have been degraded. The relative transcriptional levels of *doeA*, *doeB*, and *doeD* with 5 g/L KNO_3_ were lower than those in samples with 1 g/L KNO_3_ (0.76-, 0.77-, and 0.64-fold, respectively) (Fig S3). These results indicated that nitrogen starvation induces the transcription of ectoine-degrading genes and that ectoine may act as a nitrogen sink for *M. alcaliphilum* 20Z under nitrogen-rich conditions. Therefore, high ectoine production in proportion to cell growth can be achieved through a continuous supply of optimized nitrogen source.

In previous studies, it was considered meaningless to grow cells to a high density for the mass production of ectoine because of ectoine degradation and cessation of production at a certain growth point [13, 22, 27]; therefore, the bio-milking process was performed at a certain growth point before ectoine degradation. However, by confirming the key components for ectoine biosynthesis and optimizing the concentration of the medium components, especially nitrogen source, ectoine degradation could be prevented and ectoine synthesis could be achieved in proportion to the increase in cell density, as shown in this study. Therefore, the findings of this study allow cells to be grow to a high density and achieve high ectoine production from methane.

### Ectoine production in proportion to cell growth by optimization of culture conditions in a Fed-batch culture

**Fig. 5:**
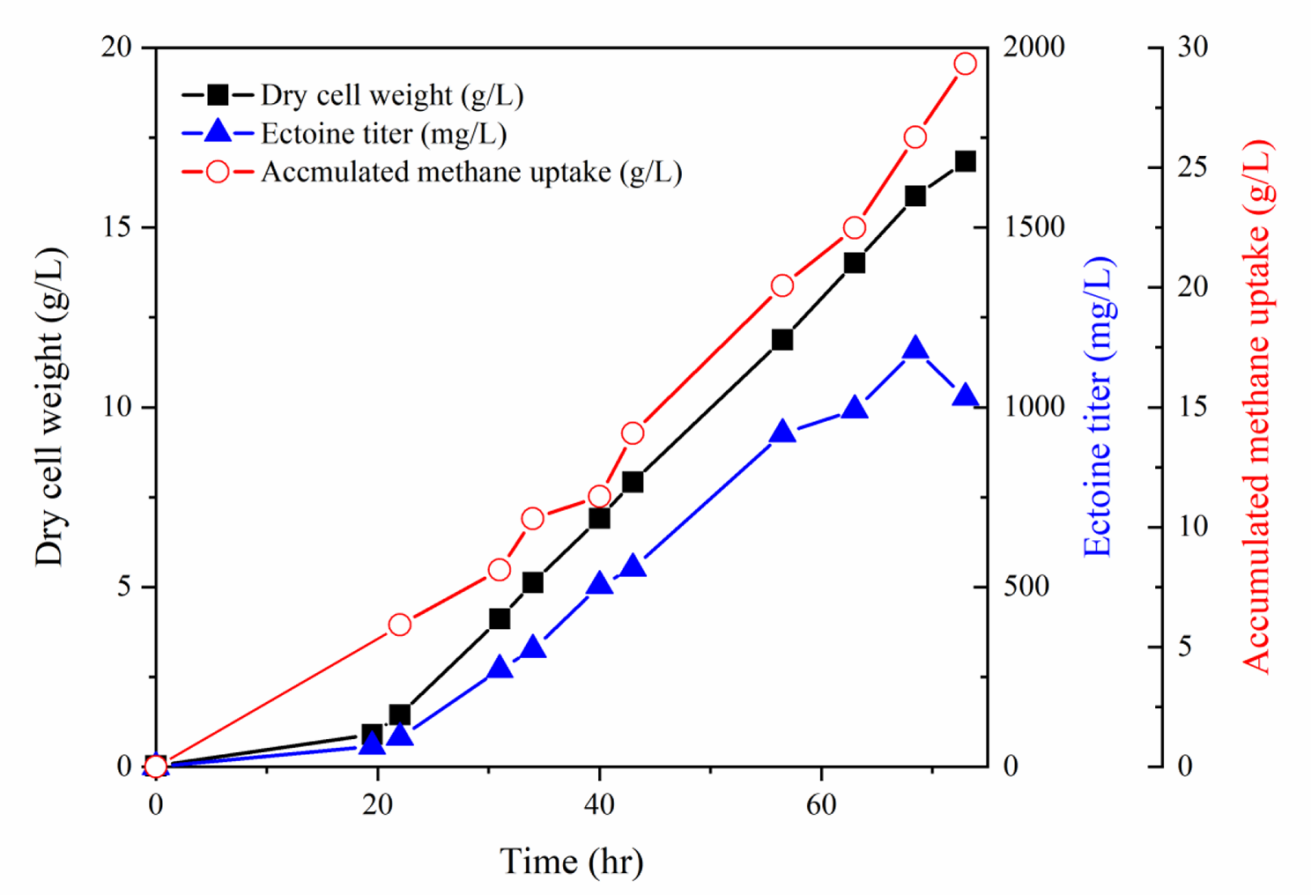
Fed-batch cultivation by *M. alcaliphilum* 20ZDP3 under optimized conditions. Fed-batch was performed with an optimized medium in a 5-L scale fermenter. For high-cell-density cultivation with a constant yield of ectoine to biomass, 2 g/L of KNO_3_ and 1X trace elements were additionally supplied every time the biomass increased by 2 g/L

To achieve high production of the ectoine, fed-batch fermentation in optimized *Methylomicrobium* medium containing 2 g/L of KNO_3_ and 0.3 g/L CuCl_2_ was carried out with pH control (pH 8.9–9.1) in a 5-L bioreactor using *M. alcaliphilum* 20ZDP3. To prevent the depletion of nitrogen sources and cofactors, 2 g/L of KNO_3_ and 1X trace elements were additionally supplied every time the biomass increased by 2 g/L. Biomass was produced up to 16.8 g/L for 73 h with a growth rate of 0.09 µ_average_ and 0.19 µ_max_ (Fig. 5). As expected, the ectoine titer steadily increased with the concentration of cells, while maintaining a constant ectoine yield (64–78 mg/g DCW) over the entire period; finally, 1.0 g/L of ectoine was produced. To investigate the feasibility of high ectoine production by *M. alcaliphilum* 20ZDP3 without growth inhibition using higher levels of KNO_3_, 5 g/L KNO_3_ was added every time the biomass increased by 2 g/L. Here, up to 18 g/L of biomass was produced at a rate of 0.1 µ_average_ and 0.17 µ_max_; however, only 0.98 g/L ectoine was produced, maintaining the ectoine yield per g DCW (data not shown). Therefore an intermittent supply of 2 g/L KNO_3_ was confirmed to be sufficient for ectoine synthesis in proportion to the increase in cell mass, which was an efficient and economical condition for fed-batch culture. In summary, ectoine production with a steady yield as well as high–cell–density culture was successfully achieved through fed-batch fermentation by applying the proposed optimal conditions. In this fed-batch culture, we measured the organic acids expelled from the cells, but organic acids such as formate, acetate, and lactate were not detected at significant levels. This indicates that the culture conditions used in this study are highly effective at minimizing the production of by-products.

To estimate the ectoine yield from methane, online gas chromatography was used. The *M. alcaliphilum* 20ZDP3 consumed 87.9 g of methane for 73 h with a yield of 0.04 g ectoine/g methane (Fig. 5). Additionally, a yield of 0.6 g biomass/g methane was obtained under the growth conditions, with the remaining methane probably released as carbon dioxide (data not shown). In this study, only 2.3% (g/g) of supplied methane was assimilated in a 5-L bioreactor, which may be due to limited gas-liquid transfer and methanotrophic methane oxidation efficiency. Considering the significant portion of the carbon flux that is converted to non-targeted products, further studies will require bioprocess design, development of novel bioreactor that overcome the limitations of gas-liquid mass transfer, and strain engineering with enhanced carbon flux to ectoine synthesis.

### High production of ectoine by hyperosmotic shock

Ectoine is an osmoprotectant that protects cells from osmotic pressure. In previous bio-milking studies, it was possible to accumulate intracellular ectoine in a short time by subjecting cells to hyperosmotic shock [37]. Therefore, a high-density culture was performed in a 5-L bioreactor to verify this effect.

To confirm the accumulation of ectoine by hyperosmotic shock after high–cell density fed-batch culture, 50 mL of culture broth was transferred to a 250-mL flask and an appropriate concentration of NaCl and a nitrogen source were added to the broth to evaluate the optimal conditions for ectoine accumulation (6%, 9% (w/v) NaCl; 5, 10 g/L KNO_3_). The cells were cultured with shaking at 30 °C for 48 h, and harvested every 24 h to measure the amount of intracellular ectoine. The initial ectoine concentration after fed-batch fermentation was 697 mg/L, which showed a significant (2.3-fold) increase upon adding NaCl and KNO_3_ (Table 3). In particular, medium containing 6% (w/v) NaCl showed accumulation of up to 1.6 g/L ectoine without biomass increase, and no differences were observed with different concentrations of KNO_3_. When 9% (w/v) NaCl was added to the medium, ectoine accumulation and production rates were lower than those in medium containing 6% (w/v) NaCl. As sufficient ectoine was accumulated with 5 g/L KNO_3_ and 6% (w/v) NaCl, these conditions were applied for fed-batch fermentation.

**Table 3:** Ectoine titer (mg/L) accumulated by hyperosmotic shock after stationary phase by addition of 6% (w/v), 9% (w/v) of salinity and 5 g/L, 10 g/L of KNO_3_ in 250-mL flask

KNO_3_ concentration (g/L)	NaCl 6% (w/v)			NaCl 9% (w/v)		
	0 h	24 h	48 h	0 h	24 h	48 h
5	697	1593 ± 24	1473 ± 18	697	1161 ± 45	1352 ± 41
10		1546 ± 27	1572 ± 29		1103 ± 36	1326 ± 68

**Fig. 6:**
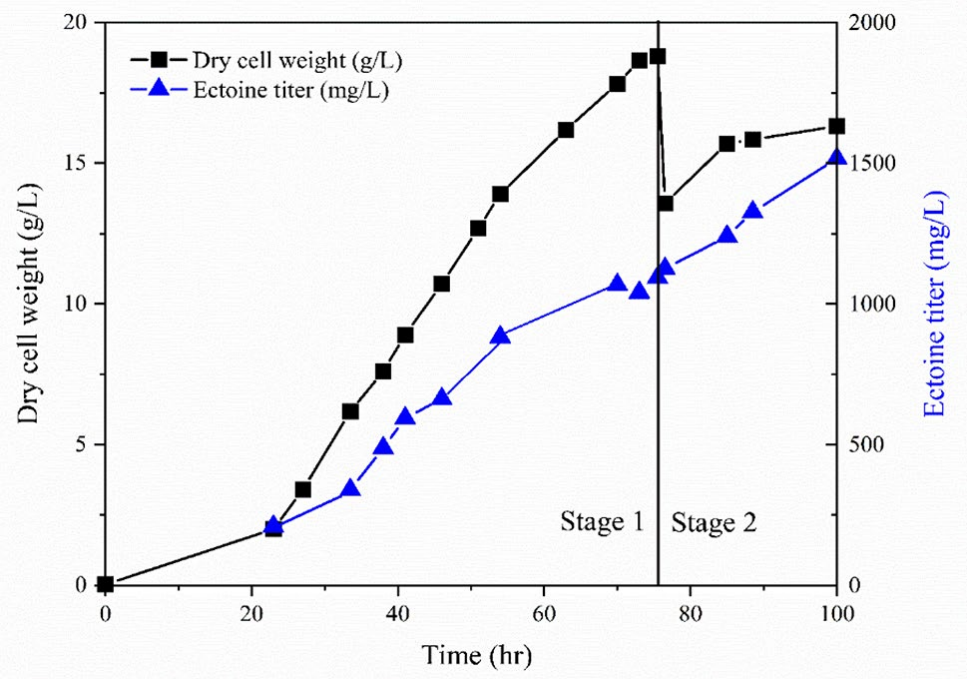
Two-stage cultivation of *M. alcaliphilum* 20ZDP3 under optimized conditions for high cell density (stage 1) and ectoine accumulation (stage 2). In stage 1, fed-batch culture was conducted using the optimized medium and 2 g/L of KNO_3_ and 1X trace elements were supplied additionally every time the biomass increased by 2 g/L until the cells reached a stationary phase. In stage 2, 5 g/L of KNO_3_ was supplied and NaCl was added to adjust the concentration to 6% (w/v) for ectoine accumulation by a hyperosmotic shock. Time course of biomass (g/L) and ectoine (g/L) production by *M. alcaliphilum* 20ZDP3

To further improve ectoine production, two stages of fermentation were conducted in a 5-L bioreactor. In the first stage, fed-batch fermentation in optimized *Methylomicrobium* medium was carried out with pH control (pH 8.9–9.1) using *M. alcaliphilum* 20ZDP3, and 2 g/L of KNO_3_ and 1X trace elements were additionally added for every 2 g/L increase in biomass to achieve maximum possible cells density with a constant ectoine yield. In the second stage, when cells were determined to have reached stationary phase and no further cell growth was considered possible, 5 g/L of KNO_3_ was supplied, and NaCl was added to achieve a concentration of 6% (w/v) to induce intracellular ectoine accumulation by hyperosmotic shock. The gas flow rate was lowered from 0.5 vvm to 0.1 vvm, while the ratio of methane to air was kept constant at 3:7 to minimize the shear stress on the cells. The cells grew with a growth rate of 0.09 µ_average_ and 0.19 µ_max_ until the biomass reached 18.7 g/L (Fig. 6). The specific growth rate (µ) was the highest in the initial 23 h and then decreased until 54 h and remained below 0.03. Ectoine was produced rapidly at 0.9 g/L until 54 h and maintained during first stage of fermentation as the specific growth rate decreased. The point at which ectoine production slowed down owing to decrease of the specific growth rate coincided with the point at which dissolved oxygen in the culture medium rapidly decreased, remained at 0%, and then began to gradually increased (data not shown), which is an oxygen-limitation effect during fermentation. Nevertheless, with additional supplementation with KNO_3_ and NaCl in the second stage, 1.5 g/L of ectoine was accumulated, which was 1.5-fold higher than that in the first stage. The ectoine yield per biomass (mg/g DCW) was remained approximately 63 mg/ g DCW during initial 54 h but it was slightly decreased afterward (58 mg/ g DCW at 75.5 h). While biomass decreased due to cell lysis caused by hyperosmotic shock, the ectoine yield per biomass was enhanced to 93 mg/g DCW in Stage 2 of fermentation by hyperosmotic shock at 24 h.

The production of ectoine using methanotrophs is much lower than that achieved through sugar fermentation by using *Halomonas* species, *Escherichia coli*, and *Corynebacterium glutamicum* [20, 38, 39]. However, this process is meaningful as it combines with the atmospheric reduction of methane, a greenhouse gas that contributes to global warming. Table 4 compares the ectoine production from methane from previous reports and this study. Until now, Cantera et al. [37] reported the highest titer of ectoine (0.25 g/L) by *M. alcaliphilum* 20Z using the bio-milking process. We had previously demonstrated that genetically engineered *M. alcaliphilum* 20Z could produce ectoine up to 0.14 g/L with the yield of 111 mg/ g DCW from methane under batch fermentation [22]. Additionally, recent studies have suggested that mixed culture of *Methylomicrobium* species were able to produce ectoine up to 105 mg/ g DCW and 109 mg/ DCW g, respectively [13, 40]. Ngoc Pham et al. [41] reported ectoine production (38 mg/ g DCW) from methane and lignocellulose-derived sugars (glucose and xylose) by genetical modification in *M. alcaliphilum* 20Z. Previous studies on the conversion of methane to ectoine have focused on the bio-milking process with wild-type stain, which has a limitation of ectoine degradation with cell growth [13, 22, 27]. In this study, we constructed *M. alcaliphilum* 20ZDP3 by removing the *doeA* gene responsible for the first step of ectoine degradation after the cells reached a mid-exponential growth phase. Ectoine production proportional to cell growth was then achieved by supplementation with a sufficient nitrogen source, and enhanced ectoine was synthesized using a hyperosmotic shock and supplying with high salt and nitrogen after the cells reached a stationary phase. Therefore, the highest ectoine titer obtained using methane (1.5 g/L) was achieved. This result was successfully accomplished by preventing ectoine degradation in *M. alcaliphilum* 20Z. More importantly, this study established the critical role of nitrogen sources in the medium for ectoine production. Additionally, it was found that the expression of genes related to ectoine degradation is regulated by the availability of nitrogen sources in the medium. Considering that the ectoine is a high value-added product, this strain exhibits an excellent industrial and environmental benefit better than other similar methanotrophs.

**Table 4:** Comparison of ectoine production by methanotrophs

Host bacteria	Carbon sources	Culture mode	Ectoine titer (g/L)	Maximum ectoine yield (mg/ g DCW)	References
*Methylomicrobium buryatense, Methylomicrobium japanense*	Methane	Batch	-	105	[40]
*Methylomicrobium alcaliphilum* 20Z	Methane	Bio-milking	0.25	-	[37]
*Methylomicrobium alcaliphilum* 20Z	Methane, Xylose, Glucose	Batch	-	38	[41]
*Methylomicrobium alcaliphilum* 20Z, mixed haloalkaiphilic consortium	Biogas	Continuous	-	109	[13]
*Methylomicrobium alcaliphilum* 20ZDP2	Methane	Batch	0.14	111	[22]
*Methylomicrobium alcaliphilum* 20ZDP3	Methane	Fed-batch	1.50	93	This study

## Conclusions

In the present study, high ectoine production from methane was achieved using engineered *M. alcaliphilum* 20ZDP3 by preventing ectoine degradation. By combining strategies:1) disruption of *doeA* gene, which is responsible for the first step of ectoine degradation, in *M. alcaliphilum* 20ZDP2; 2) supplying nitrogen source for ectoine synthesis in proportion to cell growth; 3) fed-batch fermentation using *M. alcaliphilum* DP3 supplementing KNO_3_ and trace element for high cell density culture (HCDC); 4) hyperosmotic shock after HCDC of *M. alcaliphilum* 20ZDP3, ectoine was accumulated up to 1.5 g/L with a yield of 93 mg/g DCW. The ectoine production achieved in this study is the highest among the methane-based ectoine production using methanotrophs reported to date. Further improvements in the high-cell-density cultivation of *M. alcaliphilum* 20Z strains based on this study can facilitate eco-friendly and economical processes for ectoine production by methanotrophs, beyond the currently expensive sugar-based fermentation using microorganisms.

### Electronic supplementary material

Below is the link to the electronic supplementary material.

Supplementary Material 1

## Data Availability

No datasets were generated or analysed during the current study.
