# Supplementary material for: High production of ectoine from methane in genetically engineered Methylomicrobium alcaliphilum 20Z by preventing ectoine degradation
Source: Microb Cell Fact. 2024 May 2;23:127. doi: 10.1186/s12934-024-02404-2 (PMC11067125; doi:10.1186/s12934-024-02404-2)
Supplement: Supplementary file 1 — Supplementary Material 1 [file 12934_2024_2404_MOESM1_ESM.docx]

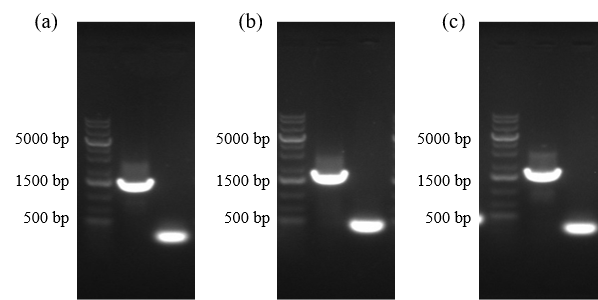


Figure S1. PCR for confirmation of a gene deleted mutants. (a) PCR for confirmation of knocking out *doeA* gene. Lane 1, size marker ; Lane 2, 1490 bp, *M. alcaliphilum* 20Z DP2; Lane 3, 314 bp, *M. alcaliphilum* 20Z DP3 popped out *doeA* gene. (b) PCR for confirmation of knocking out *doeD* gene. Lane 1, size marker ; Lane 2, 1737 bp, *M. alcaliphilum* 20Z DP2 ; Lane 3, 463 bp, *M. alcaliphilum* 20Z DP4 popped out *doeD* gene. (c) PCR for confirmation of knocking out *ectB*. Lane 1, size marker ; Lane 2, 1682 bp, *M. alcaliphilum* 20Z DP2; Lane 3,350 bp, *M. alcaliphilum* 20Z DP5 popped out *ectB*.

**

**

Figure S2. The main component profiles, such as K^+^, Mg^2+^, NO^3-^ and PO_4_^3-^, by *M. alcaliphilum* 20ZDP3. *M. alcaliphilum* 20ZDP3 was cultured in a medium for 120 h and main component of medium was analyzed by ion chromatography.





Figure S3. Comparison of transcriptional level of ectoine degradable genes of *M. alcaliphilum* 20ZDP2 cultivated in Methylomicrobium medium containing 1 g/L or 5 g/L of KNO_3_. *M. alcaliphilum* 20ZDP2 was cultivated in 1 g/L or 5 g/L of KNO_3_ containing medium and bacterial cells were collected at 72 hours.
